# The Advancement of Waterjet-Guided Laser Cutting System for Enhanced Surface Quality in AISI 1020 Steel Sheets

**DOI:** 10.3390/ma17143458

**Published:** 2024-07-12

**Authors:** Muhammed Paksoy, Hakan Çandar, Necip Fazıl Yılmaz

**Affiliations:** 1 Department of Mechanical Engineering, Engineering Faculty, Gaziantep University, Sehitkamil, 27310 Gaziantep, Türkiye; candar@gantep.edu.tr (H.Ç.); nfyilmaz2@gantep.edu.tr (N.F.Y.); 2 Board of Trustees, Hasan Kalyoncu University, 27410 Gaziantep, Türkiye

**Keywords:** fiber laser, laser cutting, waterjet-guided laser technology, surface integrity

## Abstract

This study investigates the effects of a water-guided laser on the cutting performance of AISI 1020 steel sheets of various thicknesses by comparing the results with respect to a conventional laser. For this purpose, a novel nozzle design has been devised enabling the delivery of laser beams to the workpiece conventionally as well as through water guidance. Diverging from prior literature, a fiber laser is used with a high wavelength and a laser power output of 1 kW. Experiments are conducted on steel sheets with thicknesses ranging from 1.5 mm to 3 mm using three different cutting speeds and laser power levels. Analysis focuses on assessing surface roughness, burr formation and heat effects on the cut surfaces for both conventional and waterjet-guided cutting. Surface roughness is evaluated by using a 3D profilometer and cut surfaces are examined through SEM imaging. The results showed that the waterjet-guided laser system greatly reduced surface roughness and minimized problems associated with traditional laser cutting such as kerf, dross adherence and thermal damage. The study revealed that cutting speed had a greater effect on surface roughness reduction than laser power, with the most noticeable differences occurring in thinner sheets. Furthermore, the investigation suggests that the waterjet-guided laser cutting system demonstrates superior performance relative to conventional methods, particularly in surface quality.

## 1. Introduction

In recent days, with the influence of developing technology, modern manufacturing methods are widely used in many industrial and technological fields. In order to be strong against global competition and to reach big markets, the production costs must be low; the manufacturing processes must be fast and have a high quality. Conventional manufacturing methods do not adequately cope with the goals to be achieved in this fast and technological race [1]. Depending on the technological and scientific developments, modern and automation-based manufacturing methods have begun to play an important role in closing this deficiency. Among these new and modern manufacturing methods, laser-assisted manufacturing technology becomes one of the most preferred methods in the industrial area, especially in high-economic and added-value sectors such as medical, automotive, aerospace and microelectronics [2,3].

Laser cutting provides many advantages on precision cutting of even composites, ceramics and difficult to machine materials [4]. Laser cutting does not cause any mechanical stress, rubbing and abrasion due to the fact that there is no direct contact between the tool and the workpiece compared to the other non-traditional processes [5]. In addition, it has an important place in the industry, especially successful in cutting thin sheets because of its high-cutting speeds, easy removal of complex shapes and a high-quality surface processing [6]. Although the laser cutting method offers unique advantages, the high-heat energy emitted by the laser can cause microstructural issues on the contact surfaces of the workpiece, including local flow stress problems, hardness loss, thermal breakage and local collapse. These issues can lead to unforeseen errors and design flaws, particularly in applications where mechanical properties are critical. Furthermore, the inability to direct laser beams linearly onto the workpiece results in problems such as kerf taper, high dross adhesion, poor cut surface quality and focus adjustment challenges. The necessity to adjust the focus based on material type, cutting speed and thickness, along with the requirement for consistent part thickness, further complicates and restricts the application of this method. To address these challenges, solutions have been proposed to improve laser beam direction and minimize heat-affected zones on the cutting surfaces. One effective approach called water-guided laser or laser microjet is to guide the laser through water, which can linearly direct the laser beams and simultaneously cool the surfaces during the cutting process. This method has shown promise in mitigating the key issues associated with laser cutting [7,8,9,10,11,12]. Bernold Richerzhagen, highlighting its potential to enhance laser cutting performance. In this study, the design of the laser microjet was presented and the effects of water-cooling effect, laser cutting distance and waterjet pressure on the cutting ability were investigated [13,14]. Ikeda et al. [15] used a waterjet-guided laser for cutting wafer. Wafer was cut by using a conventional laser and a waterjet-guided laser. Cutting quality of these two methods was compared. Waterjet-guided laser cutting provided less structural changes, no drawing, clean cutting edges, narrow beam diameter and slightest mechanical load during wafer cutting processing. Porter et al. [16] designed a hybrid laser and waterjet system to mitigate the thermal effects of the laser and remove cutting particles from the workpiece. The study aimed to determine the focusing distance of waterjet-guided laser. The reflection of the laser beam in the high-pressured water wall provided longer cutting distances compared to normal laser processing. It is conducted cutting operations by controlling cutting distance, cutting speeds and angle of incidence. Suvradip et al. [17] studied the development of a waterjet-assisted underwater laser cutting process. This cutting process produced less turbulence and gas bubbles compared to a gas-assisted underwater laser cutting process. Qiao et al. [18] discussed waterjet-guided laser micromachining for working with hard, brittle materials. An experiment was conducted to explore the effects of waterjet-guided laser micromachining on surface topography changes, aspect ratio evolution and kerf edge variations using an Nd: DPSS laser system with specific parameters. The relationship between machining parameters and surface changes is discussed, revealing that the technique yielded no oxidation or cracks on the kerf edge and achieved a high aspect ratio. Liu et al. [19] elaborated on the principles, machine configurations and benefits of waterjet-guided laser technology, discussing aspects such as waterjet formation, optical characteristics and factors affecting jet stability. The study displayed waterjet-guided laser-matter interaction, material removal modelling and laser-induced phenomena like bubbles and breakdown. Additionally, this advancement poses challenges in stabilizing the waterjet due to the intricate interaction between gas and water, laser parameters and the design of the coupling head. Higher laser-power intensity, while beneficial for cutting, introduces nonlinear absorption in water, leading to energy loss. Research efforts are required to address these complexities and optimize cutting processes. Overall, it provides a comprehensive comparative literature survey on waterjet-guided laser technology. An optical geometric model of the lens by Chuang et al. [20] was established through mathematical calculations, showing that the laser beam can generate multiple focal points with varying lengths. Sundar et al. [21] aimed to investigate WJG laser drilling of acute angular holes on coated nickel super alloy, comparing it with QCW millisecond fiber laser technology. Experimental analyses were conducted to assess TBC delamination, recast layer formation, hole surface topology and cycle time for both techniques. A finite element analysis-based numerical model was also developed to understand heat propagation and material removal mechanisms. Subasi et al. [22,23] conducted experiments with various hole diameter values to investigate the process limits and find out the machining efficiency depending on the hole depth for a turbine. A real-time measurement approach based on acoustic signals is proposed. Experimental results reveal a correlation between ultrasound emissions and material removal, dependent on hole geometry, in waterjet-guided laser drilling for aerospace alloys.

In light of existing literature which discusses various aspects of waterjet-guided laser cutting technology, this study aims to further explore the effects of guiding the laser through water on cutting performance. To achieve this goal, an experimental study is employed comparing traditional dry cutting with waterjet-guided cutting techniques using the same nozzle. Through this comparison, the distinct advantages offered by waterjet-guided laser cutting are aimed to be clarified. Furthermore, the experimental design includes an examination of variations in material thickness, as steel sheets of different thicknesses are analyzed simultaneously. Within this framework, the key parameters of laser cutting, such as cutting speed and laser power, are systematically varied and examined to reveal their impact on cutting quality. Additionally, the potential of fiber lasers as water-guided tools is explored; thereby, pushing the boundaries of current technological capabilities.

## 2. Materials and Methods

**Figure 1:**
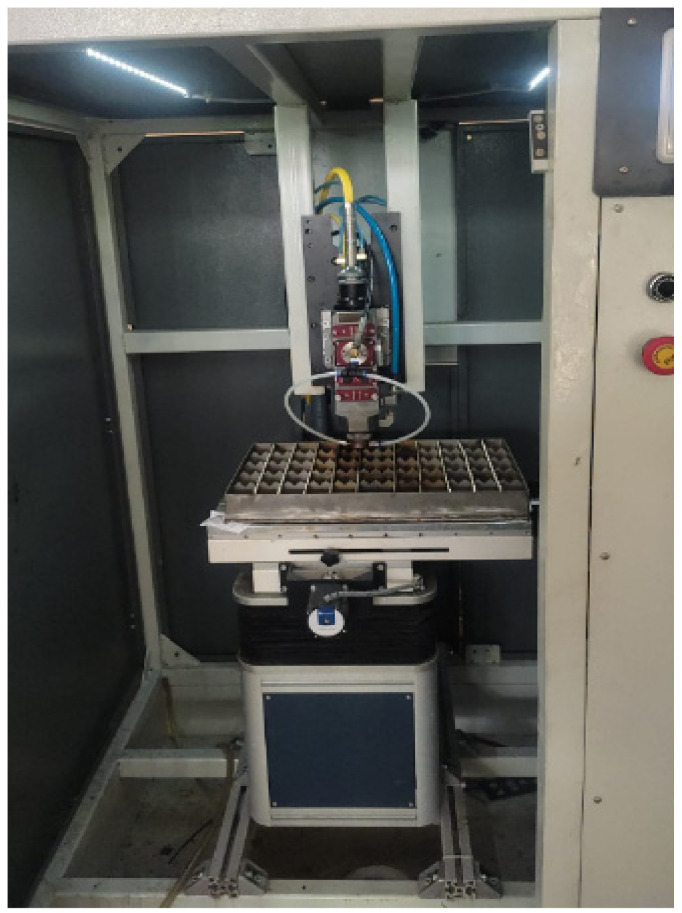
Design of waterjet-guided laser unit.

Cutting processes are performed on the laser cutting unit shown in Figure 1. The unit is CNC-controlled and facilitates movement in three axes. In order for the laser beams to be guided through water, the wavelength of the laser must pass through the water transmission spectrum [24]. For this reason, high wavelength ND:YAG lasers have generally been used in the literature. In this study, a fiber laser with a high wavelength (1080 nm) was used. The laser has a maximum power of 1 kW, focusing length of 125 mm, collimation length of 100 mm and the maximum pulse repetition frequency of 20,000 Hz. A nozzle with a 1 mm spot diameter was designed that can provide both normal and water-guided cutting processes, and a stand-off distance was set to 20 mm during the water-guided cutting operations [16]. A pump system is utilized to deliver water to the laser cutting head. The water required for the process is filtered and supplied to the cutting head from the mains. The flow rate of water in the pump can be adjusted, but a constant flow rate of 2.25 m^3^/s was used in the experiments.

**Figure 2:**
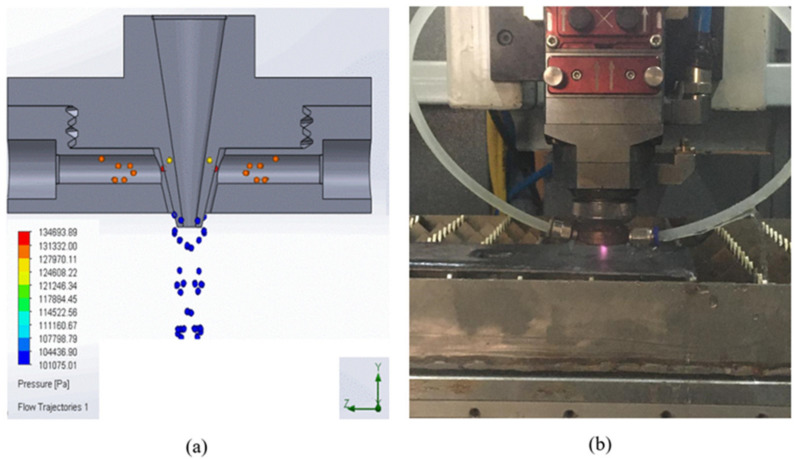
(**a**) Modelling of cutting head and water flow. (**b**) Waterjet-guided laser head.

In order to guide the laser in the cutting head, various nozzle designs and flow analyses are conducted in the SolidWorks 3D modelling program. The aim of the analyses is to ensure that the flow of water towards the workpiece is vertical and with low turbulence. Additionally, to maintain the laser focus, the length of the nozzle is set equal to the laser’s own nozzle length. Taking these factors into account, several samples are produced and tested. Figure 2 illustrates the flow analysis of the nozzle geometry that provides the best flow of water from the laser head during the cutting process.

**Figure 3:**
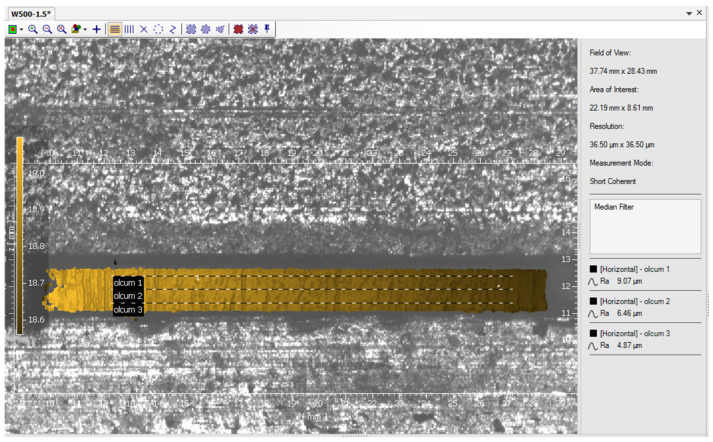
Measurement method of surface roughness.

Sheet thickness, cutting speed and laser power parameters are utilized to understand the operating conditions of the water-guided laser cutting system created within the scope of the study and to identify the ideal conditions for cutting AISI 1020 steel sheets of various thicknesses. AISI 1020 steel sheets with different thicknesses, such as 1.5 mm, 2 mm, 2.5 mm and 3 mm, are cut under different parameters with the waterjet-guided laser cutting system. Due to 1 kW laser power capacity and usage of waterjet-guided laser for machining and micromachining, small sheet thicknesses were considered in the experiments. Oxygen gas (99.95% purity) pressure is established before beginning the cutting operations based on testing to find the optimal pressure for each sheet of AISI 1020 steel. Because the developed water hybrid laser cutting nozzle does not work under standard laser cutting circumstances when water travels through it, adequate pressure values for each layer are determined. Oxygen gas pressures of 2.5 Bar, 3 Bar, 5 Bar and 7 Bar are set for AISI 1020 steel sheets with thicknesses of 1.5 mm, 2 mm, 2.5 mm and 3 mm, respectively. All experimental conditions are applied independently to conventional laser and waterjet-guided laser cutting processes. Laser power between 100 W and 1000 W is delivered at 100 intervals for all thicknesses after the oxygen gas pressures are ascertained. Cutting is done at four distinct speeds: 15 mm/s, 20 mm/s, 25 mm/s and 30 mm/s, using each laser power. At any thickness utilized in the experiment, cutting below 400 W is not possible. The samples are cut with a nozzle produced without the use of water in order to compare the ones cut at laser powers of 400 W and above. The cut samples were investigated by using optical profilometers such as the Polytec TMS100 3D profilometer, according to ISO 4287-4288 standards [25]. In the measurements, average roughness (Ra) values were taken along three different lines on the surface as seen in Figure 3, and the average was calculated. The lines were drawn 0.25 mm below the top and 0.25 mm above the bottom edges of the specimens as seen in Figure 1. Scanning electron microscopy (SEM) images are also taken from the SEM (Gemini 300, Zeiss, Oberkochen, Germany) in order to compare the conventional laser cut surface and waterjet-guided laser cut surface in means of burr formation.

## 3. Results and Discussion

In the investigation of surface roughness values between conventional laser cutting and waterjet-guided laser cutting, Table 1 reveals distinct differences. The surface roughness values, denoted as “L” for conventional laser cutting and “WGL” for water-guided laser cutting, highlight the impact of the cutting method on surface quality. In order to make a comparison for all samples that could be cut safely with the waterjet-guided laser cutting method, cutting was carried out under the same conditions with a traditional fiber laser. The roughness values that are not displayed in the table are from samples that the waterjet-guided laser was unable to cut. The lowest values for sheet plates with thicknesses of 1.5 mm, 2 mm, 2.5 mm and 3 mm were 0.937 µm, 0.630 µm, 1.210 µm and 1.990 µm in the surface roughness tests conducted with an optical profilometer. These results are stated in bold font in Table 1. Under the same settings, samples cut with a conventional fiber laser showed a notable variation in surface roughness according to specimens produced by the waterjet-guided laser cutting system. The greatest difference between the two cutting methods was seen at 1.5 mm, but it was observed that the difference between the surface roughness from the use of the conventional laser and waterjet-guided laser decreased as the thickness increased. However, it was observed that there was a difference of approximately five times between the samples obtained from the two cutting methods at most, and a difference of approximately two times in the lowest one.

**Figure 4:**
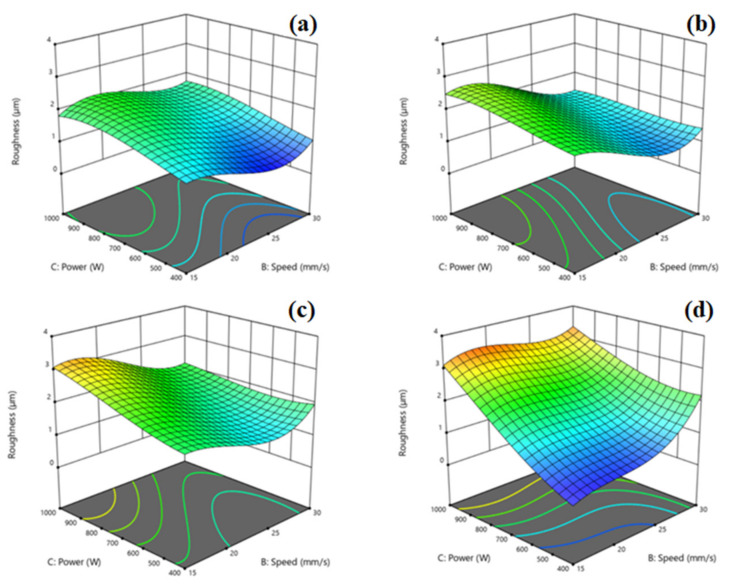
Response surface 3D plots for the effects of independent variables of cutting speed (B) and laser power (C) on surface roughness for waterjet-guided laser cutting. (**a**) 1.5 mm thickness, (**b**) 2 mm thickness, (**c**) 2.5 mm thickness, (**d**) 3 mm thickness of AISI 1020 steel sheet.

Figure 4 shows the response surface 3D plots of roughness variations at various thicknesses as a function of laser power and cutting speed. The 3D surface plot of the 1.5 mm thick AISI 1020 sheet plate is given in Figure 4a. It was observed that the roughness values decreased relatively as the laser power decreased. At the same time, it was observed that there was an improvement in roughness at very low levels with the increase of cutting speed. It was found that the cutting speed had bigger influence than the laser power. Similar results were obtained for the 2 mm thick sheet, as seen in Figure 4b. On the other hand, the trends related to cutting speed and laser power were found to be similar in both the 2 mm and 2.5 mm specimens, as observed in Figure 4b,c. It was found that samples with a thickness of 3 mm showed a distinct trend compared to the other three 3D plots, as given in Figure 4d. In samples with a thickness of 3 mm, it was found that cutting speed and laser power had a more significant impact on surface roughness. It was found that samples with a thickness of 3 mm showed a distinct trend compared to the other three 3D plots. In samples with a thickness of 3 mm, it was found that cutting speed and laser power had a more significant impact on surface roughness. Another noteworthy circumstance is that, in contrast to other sample groups, cutting speed was more efficient here, but the trend’s direction had also changed. For thicknesses of 1.5 mm, 2 mm and 2.5 mm, the surface roughness generally decreased as the speed increased. However, for a thickness of 3 mm, the roughness was found to be at its lowest level at a speed of 15 mm/s, which was the lowest speed tested. It was thought that the reason for the change in the trend in the roughness results due to 3 mm thick cuts was that the nozzle design developed with the increase in thickness made cutting more difficult at high speeds. In this regard, it has been shown in the literature that in scenarios with a fixed laser capacity, slow speeds may be chosen over high speeds after a certain thickness [26]. The results of the experiment show similarity that the laser power exerted significant influence on cutting quality. This indicates that the cutting speed also played a role in influencing roughness. A higher cutting speed led to a shorter cooling effect from the waterjet on the material, resulting in severe ablation of the material surface. Conversely, reducing the cutting speed allowed the waterjet to interact with the material surface for a longer duration. Consequently, more heat could be dissipated during the laser pulse; thereby, decreasing heat transfer and accumulation within the material [27].

**Figure 5:**
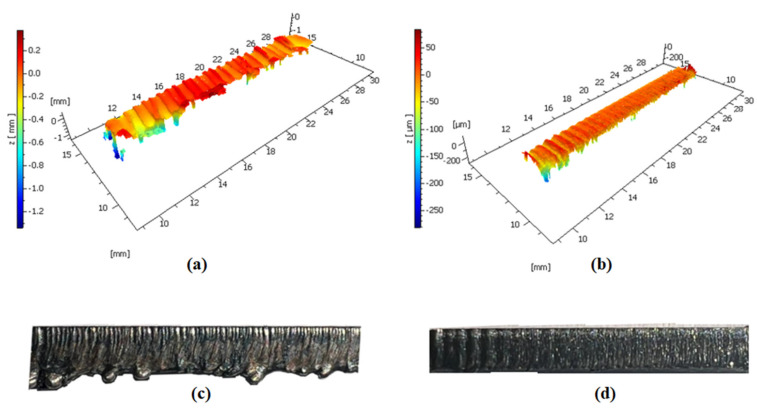
3D topography and macro view of the surface cut by (**a**,**c**) conventional laser and (**b**,**d**) water-guided laser, respectively.

The example of macro and 3D surface topography images for conventional and waterjet-guided laser cut samples are given in Figure 5. The contrast in local color between valleys and peaks in 3D surface topography images can be used to compare the surface quality of different surfaces. It is evident that there were noticeable changes in the surface textures depending on the cutting methods used. It is expected that the distorted surface resulted from excessive laser power used in the traditional laser method. Based on both macro and 3D surface topography images, it is evident that waterjet-guided cutting eliminates the distorted and irregular surfaces encountered in traditional cutting methods under the same conditions. Additionally, a significant difference was detected in burr formation between the conventional and waterjet-guided laser cut samples. As anticipated, a negligible burr formation occurred during the waterjet-guided laser cutting procedure. However, because of the high-laser power using in the conventional laser cutting process in order to compare the waterjet-guided laser cutting process in the same conditions, rough burr formation appeared on the surface. This was interpreted as indications that, despite the created waterjet-guided nozzle’s ability to cut high-laser powers, burr formation was not significantly facilitated by it. This advantage arising from the burr formation was also observed in previous studies on waterjet-guided laser [23,24].

**Figure 6:**
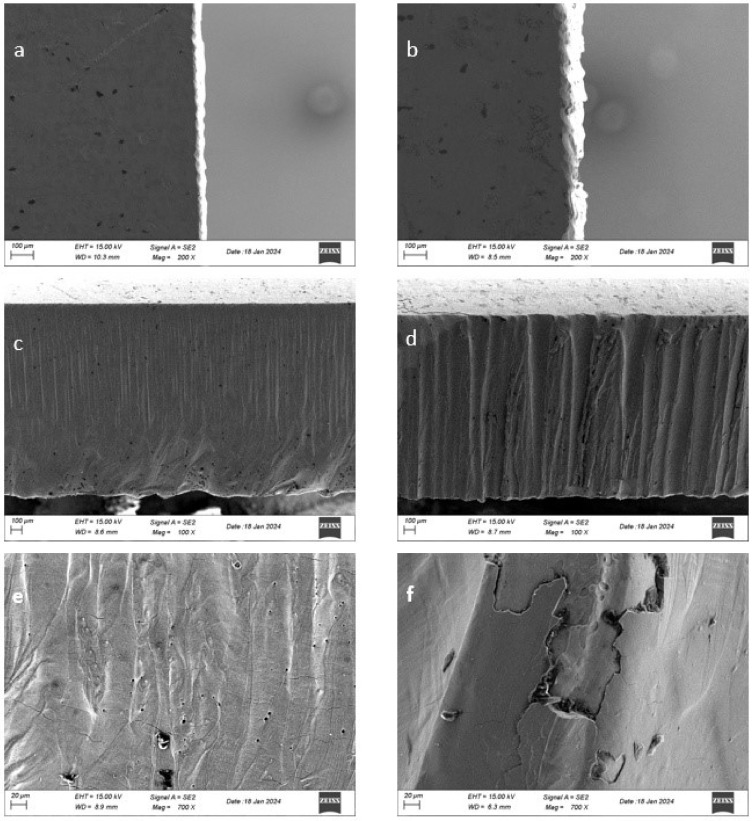
SEM morphologies of top cutting surfaces by (**a**) waterjet-guided laser and (**b**) conventional laser; SEM morphologies of cutting surface by (**c**) waterjet-guided laser and (**d**) conventional laser; zoomed SEM morphologies of cutting surface by (**e**) waterjet-guided laser (**f**) conventional laser.

Figure 6 depicts SEM images of two samples with a thickness of 2 mm, cut with waterjet-guided and traditional fiber laser methods at a speed of 30 mm/s and a laser power of 600 W. In this figure, Figure 6a,c,e shows images cut with a waterjet-guided fiber laser, Figure 6b,d,f is SEM images of samples cut with the conventional laser cutting system. In order to thoroughly and clearly examine and understand the processing traits of two laser processing techniques, SEM images of surface morphology were captured from three distinct perspectives within the images: the cut surface, the top surface (to assess the quality of the upper edge of the cut more thoroughly) and the bottom surface (to assess the quality of the lower edge of the cut completely). In Figure 6a,b, it is seen that white layer formation decreases in water-guided laser cutting. This is because water simultaneously cools the cutting surface during the cutting process. On the surfaces taken from the cutting surface (Ref. Figure 6c,d), it was observed that the water-guided laser significantly reduced the waviness on the surface. The reason for this is interpreted as the laser power being distributed more evenly on the part surface. Finally, it was revealed in Figure 6e,f that the microstructure is more regular and thinner for water-guided laser cutting with respect to conventional laser cutting.

In order to define the effects of the parameters on surface roughness for water-guided laser cutting in a statistical way, analysis of variance (ANOVA) is also employed, as shown in Table 2. The cubic effects of sheet metal thickness (A), cutting speed (B) and laser power (C) were examined using the surface roughness model. It was obtained that the proposed model fits the experimental data well and that independent parameters have significant effects on the responses from the results of ANOVA. Among the significant terms, the interaction term of B and A^2^B has the most significance with very small *p* values (*p* < 0.05). The following terms were detected as significant in the relationship between surface roughness and independent factors such as cutting speed (B) and the squared (second-order) term (A^2^B). Other terms were not found as statistically significant from the ANOVA results.

Surface Roughness (µm) = 1.88 + 0.3539A − 0.9926B + 0.5695C + 0.2876AB + 0.3277AC − 0.1837BC − 0.2140A^2^ + 0.1803B^2^ + 0.0266C^2^ − 0.2167ABC + 0.6074A^2^ B + 0.4684A^2^ C + 0.0853AB^2^ + 0.2227AC^2^ − 0.2859B^2^ C + 0.0580BC ^2^ − 0.2741A^3^ + 0.5434B^3^ − 0.1202C^3^ (1)

Equation (1) presents a surface roughness prediction model for waterjet-guided laser cutting in terms of coded variables. Additionally, it was determined that the most effective individual factor was cutting speed, followed by laser power and sheet thickness, respectively.

## 4. Conclusions

In this study, both conventional and water-guided laser cutting processes were performed using a novel nozzle design. This allowed for a detailed examination of the effects of cutting parameters on cutting performance and surface quality using the same laser unit. The primary cutting factors, laser power and cutting speed, were experimentally analyzed for both cutting methods across different ranges, and their impacts on surface quality were investigated. Additionally, the influence of material thickness on the outcomes was studied. For AISI 1040 material, the upper and lower limits of cutting parameters were determined in relation to material thickness for both cutting speed and laser power. Consequently, no specific experimental design method was employed in this study. The findings obtained from this research are summarized as follows:

- Water-guided laser cutting resulted in a significant reduction in surface roughness compared to conventional laser cutting.
- Laser power was found as a crucial parameter in performing the water-guided laser cutting process, and cutting of AISI 1040 material did not occur at powers below 400 W. Furthermore, laser power was observed to significantly impact surface quality in both cutting methods, generally having a detrimental effect on cutting quality.
- Cutting speed was identified as another influential parameter, exhibiting different behaviors based on material thickness. A decrease in surface roughness was observed with increasing cutting speed for material thicknesses between 1.5 mm and 2.5 mm, whereas an increase in surface roughness was noted for a material thickness of 3 mm with higher cutting speeds.
- Macro and surface topography analyses revealed that water-guided laser cutting substantially reduced burr formation compared to conventional laser cutting.
- SEM images indicated that water-guided laser cutting produced a smaller white layer formation and notable improvements in surface waviness compared to conventional laser cutting. Additionally, the microstructure of the cutting surface was more regular and finer in structure.
- An ANOVA analysis was conducted based on the surface roughness obtained in the study for the water-guided laser cutting process. The statistical analysis provided insights into the effects of cutting parameters and material thickness on surface roughness.
- The study also concluded that fiber lasers, due to their high wavelength, can be effectively utilized for water-guided laser cutting processes.

## Figures and Tables

**Table 1:** Comparison of average surface roughness (Ra) results depend on sheet thickness, cutting speed and laser power.

			1.5 mm		2 mm		2.5 mm		3 mm	
			Ra (µm)		Ra (µm)		Ra (µm)		Ra (µm)	
Exp. No	Cutting Speed [mm/min]	Laser Power [W]	L	WGL	L	WGL	L	WGL	L	WGL
1	15	400	3.365	1.045	3.525	2.047				
2	15	500	4.292	1.342	3.905	2.645				
3	15	600	5.532	1.707	5.245	2.665	3.685	1.752		
4	15	700	2.951	1.773	5.575	2.962	3.791	1.965	4.161	2.160
5	15	800	3.552	1.342	3.855	2.284	3.767	2.172	4.082	2.157
6	15	900	3.927	1.912	4.337	3.694	5.421	2.152	4.651	2.705
7	15	1000	3.432	2.035	3.482	2.205	3.855	2.835	4.480	3.467
8	20	400	3.573	1.007	4.365	1.392				
9	20	500	3.542	1.252	4.907	2.062				
10	20	600	5.025	1.647	4.502	2.105	3.867	**1.210**		
11	20	700	5.195	1.165	4.165	2.414	4.205	1.930	6.107	2.082
12	20	800	5.337	2.054	6.035	3.212	4.857	2.137	5.362	2.187
13	20	900	3.545	1.685	4.957	3.572	4.347	2.981	4.627	2.685
14	20	1000	4.213	1.992	4.777	2.614	4.912	2.281	4.085	3.391
15	25	400	3.932	**0.937**						
16	25	500	2.785	1.225	4.965	1.192				
17	25	600	2.702	1.265	5.197	0.965	3.732	1.377		
18	25	700	3.312	1.517	4.832	0.911	3.705	2.202		
19	25	800	2.922	1.802	4.325	0.665	3.844	2.802	3.130	2.072
20	25	900	4.558	1.525	3.654	0.835	2.917	1.875	3.072	2.692
21	25	1000	5.515	1.972	5.757	1.647	3.052	2.945	3.591	3.297
22	30	400								
23	30	500	3.187	1.017						
24	30	600	3.335	1.095	1.735	**0.630**	3.935	2.472		
25	30	700	3.054	2.002	1.965	1.227	3.645	2.150		
26	30	800	3.457	2.057	2.872	0.965	3.515	3.112	4.122	**1.990**
27	30	900	3.612	2.092	2.332	1.037	4.297	2.610	4.502	2.602
28	30	1000	3.182	1.817	3.127	0.812	3.642	2.422	3.460	3.242

**Table 2:** Model summary and analysis of variance (ANOVA) with response of surface roughness for waterjet-guided laser.

Source	Sum of Squares	df	Mean Square	F-Value	*p*-Value	
**Model**	28.06	19	1.48	5.38	<0.0001	significant
**A-Material Thickness**	0.3770	1	0.3770	1.37	0.2455	
**B-Cutting Speed**	3.10	1	3.10	11.28	0.0013	
**C-Laser Power**	1.03	1	1.03	3.76	0.0569	
**AB**	0.7397	1	0.7397	2.69	0.1055	
**AC**	0.1458	1	0.1458	0.5309	0.4688	
**BC**	0.1477	1	0.1477	0.5378	0.4659	
**A²**	0.2210	1	0.2210	0.8048	0.3729	
**B²**	0.3876	1	0.3876	1.41	0.2390	
**C²**	0.0015	1	0.0015	0.0053	0.9420	
**ABC**	0.1778	1	0.1778	0.6476	0.4239	
**A²B**	2.67	1	2.67	9.72	0.0027	
**A²C**	0.4428	1	0.4428	1.61	0.2086	
**AB²**	0.0557	1	0.0557	0.2030	0.6538	
**AC²**	0.0703	1	0.0703	0.2560	0.6146	
**B²C**	0.3790	1	0.3790	1.38	0.2442	
**BC²**	0.0103	1	0.0103	0.0376	0.8469	
**A³**	0.2278	1	0.2278	0.8296	0.3657	
**B³**	1.00	1	1.00	3.65	0.0604	
**C³**	0.0323	1	0.0323	0.1178	0.7326	
**Residual**	18.12	66	0.2745			
**Cor Total**	46.18	85				

## Data Availability

The original contributions presented in the study are included in the article, further inquiries can be directed to the corresponding author.
